# AI–Enabled Interpretation Guidance for Hemostasis Testing with TEG^®^ 6s

**DOI:** 10.3390/diagnostics16172852

**Published:** 2026-09-04

**Authors:** Jan Hartmann, Dana Souter, Joao D. Dias, Qun Sha

**Affiliations:** Haemonetics Corporation, 125 Summer Street, Boston, MA 02110, USA

**Keywords:** AI-driven interpretation, MetaTEG, thromboelastography, viscoelastic hemostatic assays, qualitative scoring, clinical decision support, artificial intelligence, AI

## Abstract

**Background/Objectives**: The TEG^®^ 6s is a hemostasis analyzer system used to assess viscoelastic properties of whole blood. Although it provides rapid and comprehensive insights, interpreting thromboelastography tracings requires robust clinical training and is not standardized. Our aim was to develop and evaluate a prototype of MetaTEG—an AI-driven support tool for interpreting TEG^®^ 6s tracings, with a focus on the Global Hemostasis-Heparin Neutralization cartridge. **Methods**: TEG^®^ 6s tracings, obtained from Haemonetics Corporation’s internal case library, were used to train a custom AI agent developed with Microsoft Copilot Studio. Five different clinical cases were used for testing. The AI agent incorporated knowledge graph augmentation and prompt engineering to integrate published literature and assess coagulation states based on R-time, maximum amplitude, and other key parameters. The evaluation of the AI virtualization outcomes included semiquantitative scoring by an internal TEG expert panel who assessed the AI’s diagnostic accuracy, tracing interpretation, and therapeutic recommendations. UI/UX prototypes were designed in Figma to demonstrate potential integration into clinical workflows. **Results**: Using this proof-of-concept dataset and qualitative expert scoring, MetaTEG accurately described and interpreted TEG^®^ 6s tracings and suggested potential treatment options. Reviewers consistently rated the AI-generated outputs as both clinically useful and accurate. **Conclusions**: The MetaTEG prototype represents the first application of generative AI for TEG^®^ 6s interpretation, showcasing the potential of AI-powered, human-in-the-loop decision support in hemostasis diagnostics.

## 1. Introduction

Viscoelastic hemostatic assays (VHAs) encompass technologies, such as thromboelastography (TEG^®^) and thromboelastometry (ROTEM^®^). VHAs are advanced diagnostic tests that provide a comprehensive, real-time picture of the blood clotting process. Rather than measuring isolated parts of the coagulation cascade like conventional tests, VHAs evaluate the overall quality of a clot, including its formation, strength, and breakdown. This allows for real-time evaluation directly at the point-of-care [1,2]. TEG^®^-guided therapy has been shown to improve patient blood management and certain clinical outcomes for patients undergoing surgery [3,4], as well as for patients with cirrhosis [5] or trauma, including brain injuries. Understanding a patient’s complete hemostasis status quickly can significantly impact clinical outcomes and the utilization of blood products. However, the interpretation of VHA systems remains challenging [6].

Indeed, there are multiple available algorithms and parameters from several assays [7]. A comprehensive TEG^®^ 6s-based treatment algorithm was developed to guide hemostatic interventions in cardiac surgery patients during or immediately after surgery [3]. However, interpreting TEG 6s tracings presents several challenges [8]. Firstly, physicians require robust training to be able to interpret the TEG tracings confidently, accurately, and quickly. Moreover, as teaching hospitals experience high physician turnover, regular re-training may also be necessary. Secondly, individual interpretation also results in a lack of standardization. Variability in interpretation may be particularly relevant in emergency or perioperative settings where rapid treatment decisions are required and where clinicians with different levels of experience may be involved in patient management. In addition, institutions may implement local protocols that differ in their interpretation thresholds or treatment recommendations, which can further contribute to inconsistencies in clinical practice.

Another significant challenge is the growing complexity of managing coagulation in critically ill and surgical patients [9]. Modern patient blood management strategies often require integrating multiple laboratory and clinical variables within a short time frame. Although TEG^®^ 6s provides rapid and information-rich outputs, clinicians must still synthesize multiple assay parameters simultaneously, including clot initiation, propagation, clot strength, and fibrinolysis. This process can be cognitively demanding, especially in high-acuity situations such as trauma resuscitation, liver transplantation, extracorporeal circulation, or major cardiovascular surgery. Consequently, there is growing interest in technologies that can augment clinician interpretation and improve consistency without replacing physician oversight [10]. There is a growing demand for healthcare decision-making tools that can provide standardized, high-quality test results without extensive need to train staff [11]. As artificial intelligence (AI) has rapidly transformed the medical field, it has the potential to make healthcare more equitable, cost-effective, and efficient [12]. Recent attempts have been made to use machine learning as an AI-assisted method for interpreting VHA [13,14]. However, a very recent Cochrane analysis has concluded that there have been no studies comparing AI-assisted transfusion protocols for severely bleeding trauma patients, and therefore there is a scientific gap that needs to be filled [9].

The recent emergence of large language models (LLMs) and multimodal AI systems has created new opportunities for clinical decision support [15]. Unlike conventional machine learning approaches, which rely solely on predefined statistical relationships, generative AI systems can interpret context and interact with natural language [16]. This capability is particularly relevant in coagulation management, where clinicians must consider numerical parameters, waveform characteristics, procedural context, and evolving treatment algorithms. Therefore, AI-enabled tools therefore have the potential to not only classify VHA patterns but also to explain findings in clinically meaningful language to support physician decision-making and education.

We designed a prototype of MetaTEG, a novel AI tool developed specifically for interpreting TEG^®^ 6s tracing. It integrates published clinical data with a natural language interface to provide contextual decision support at the point of care. MetaTEG could therefore limit the need for manual interpretation and standardize care, regardless of who is interpreting the tracing; however, as a human-in-the-loop concept, the final call on the interpretation is always performed by the treating physician. The focus of this proof-of-concept work is on the Global Hemostasis-Heparin Neutralization cartridge [17,18] which broadens the clinical scope of viscoelastic testing to heparinized patients.

The Global Hemostasis-Heparin Neutralization cartridge was chosen due to its importance in modern perioperative and critical care medicine, especially in cardiovascular surgery and extracorporeal support settings, where systemic heparinization is common. Its ability to accurately evaluate coagulation status despite the presence of heparin is an important advancement in viscoelastic testing. Furthermore, interpreting these assays can be complex because clinicians must distinguish true coagulopathy from heparin-related effects. This clinical complexity provided a suitable framework for evaluating the feasibility of AI-assisted interpretation.

In this report, we present the results of an internal proof-of-concept evaluation of MetaTEG, which was conducted by a panel of TEG experts. Our hypothesis was to test the feasibility of the MetaTEG prototype—an AI-driven clinical decision support tool—in accurately and reliably interpreting TEG^®^ 6s tracings and providing diagnostic and therapeutic suggestions at a level comparable to that of expert human interpretation. The aim is to provide actionable insights directly at the point of care.

## 2. Materials and Methods

**Figure 1:**
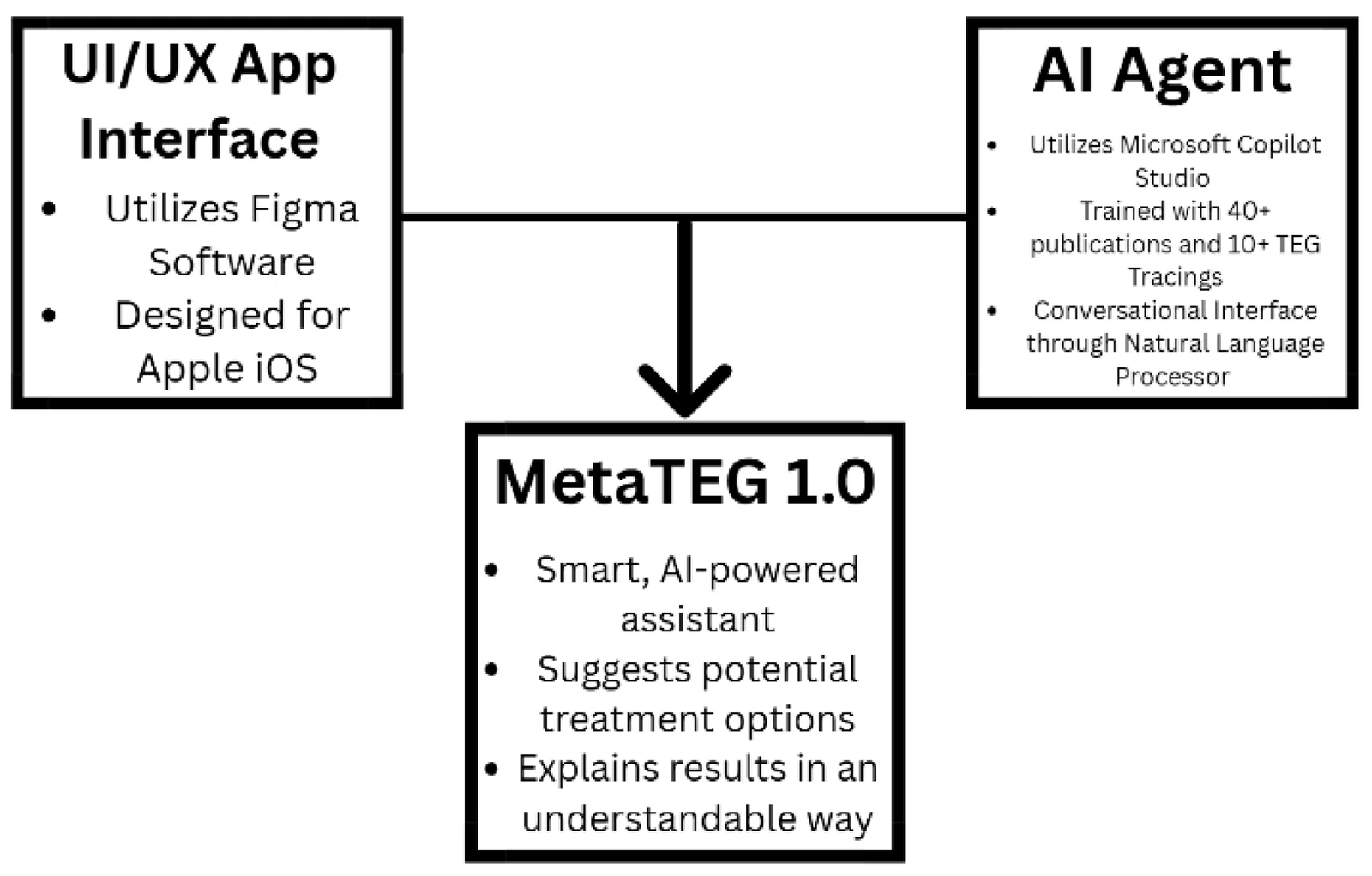
The MetaTEG version 1.0 prototype comprises two parts. AI, artificial intelligence; iOS, iPhone operating system.

Microsoft Copilot Studio (Microsoft Corporation, Redmond, WA, U.S.A.) based on GPT-4 (OpenAI, San Francisco, CA, U.S.A.) was used to develop the MetaTEG AI agent. The agent’s knowledge base was compiled from over 40 peer-reviewed publications and clinical data sources (Figure 1). The MetaTEG version 1.0 prototype was developed and validated using data from the Global Hemostasis-Heparin Neutralization cartridge of the TEG^®^ 6s hemostasis analyzer system (Haemonetics Corp., Boston, MA, USA). This cartridge can run four assays simultaneously in a single “citrated multichannel” cartridge: citrated kaolin (CK), CK with heparinase (CKH), citrated RapidTEG with heparinase (CRTH), and citrated functional fibrinogen with heparinase (CFFH).

TEG^®^ 6s tracing data were extracted from TEG Manager^®^ in image format and used for AI interpretation. The MetaTEG prototype was trained to categorize the findings as ‘normal coagulation’, ‘hypocoagulable’, or ‘hypercoagulable’ based on the manufacturer’s normal reference ranges and the most recent publications.

The AI agent uses custom knowledge libraries that include published clinical guidelines, reference ranges, and therapeutic algorithms. Using the orchestration layer in Copilot Studio, the AI agent processed these inputs via image-to-text transcription, rules-based classification, and natural language processing (NLP)-driven explanation generation. This enabled the AI agent to identify coagulation abnormalities and generate clinically relevant, contextualized recommendations. To improve the consistency of interpretations, prompt engineering techniques were implemented to standardize the structure and terminology of AI-generated output. These prompts guided the AI agent through a systematic evaluation of clot initiation, clot kinetics, clot strength, fibrinolysis, and fibrinogen contribution prior to generating diagnostic interpretations and therapeutic considerations. Additionally, contextual references from published literature and manufacturer recommendations were incorporated into the retrieval process through knowledge graph augmentation strategies. This framework aligned the model’s generated outputs with accepted clinical concepts and viscoelastic testing terminology.

The user interface of the MetaTEG prototype was developed using the Figma software (Figma, Inc., San Francisco, CA, USA) application (Figure 1). The UI/UX design and functionality were designed for clinical integration, featuring color-coded alerts, tracing summaries, and decision support dialogs tailored for both clinical and administrative users. MetaTEG uses NLP and rule-based inference engines to deliver interpretative outputs. Each AI test was prompted as follows: “Interpret TEG Tracing”, followed by the input of an image of a TEG tracing. The AI-generated interpretation was then evaluated by an internal TEG expert panel from Haemonetics’ Medical Affairs team specialized in interpreting TEG tracings. Two rounds of testing were conducted in the first week of August 2025. The first testing round included ten tracings from a variety of cartridge types, and the second round of testing included five new tracings from Global Hemostasis-Heparin Neutralization cartridges.

The second round of testing focused specifically on the heparin neutralization cartridge to evaluate the consistency of AI interpretation in a clinically relevant, technically complex setting. The selected cases included patterns representative of those encountered in perioperative and critical care medicine. These patterns included normal coagulation profiles, prolonged clotting times, impaired clot strength, and mixed coagulopathies. These scenarios were selected to challenge the model’s ability to synthesize multiple assay parameters simultaneously.

For each tracing, each reviewer was asked to rate the AI response using a scale from 0 to 10, with ‘10’ being a fully accurate response with all details included and ‘0’ a completely incorrect response. The questions were as follows: (1) ‘Was the description of the tracing accurate?’, (2) ‘Did the AI-agent provide an accurate diagnosis based on the tracing?’ and (3) ‘Did the agent offer an adequate therapeutic suggestion?’. The outcomes were presented as descriptive statistics. Standard deviation values were calculated to evaluate the variability between reviewer scores and to identify areas of strongest and weakest agreement.

The expert review process was designed to provide a semi-quantitative assessment of clinical usefulness and interpretive accuracy. Reviewers evaluated the outputs independently without discussion or consensus scoring. This methodology was not intended to establish formal diagnostic performance metrics, but rather to assess the feasibility of proof of concept and determine whether experienced TEG reviewers considered AI-generated interpretations clinically reasonable.

## 3. Results

**Figure 2:**
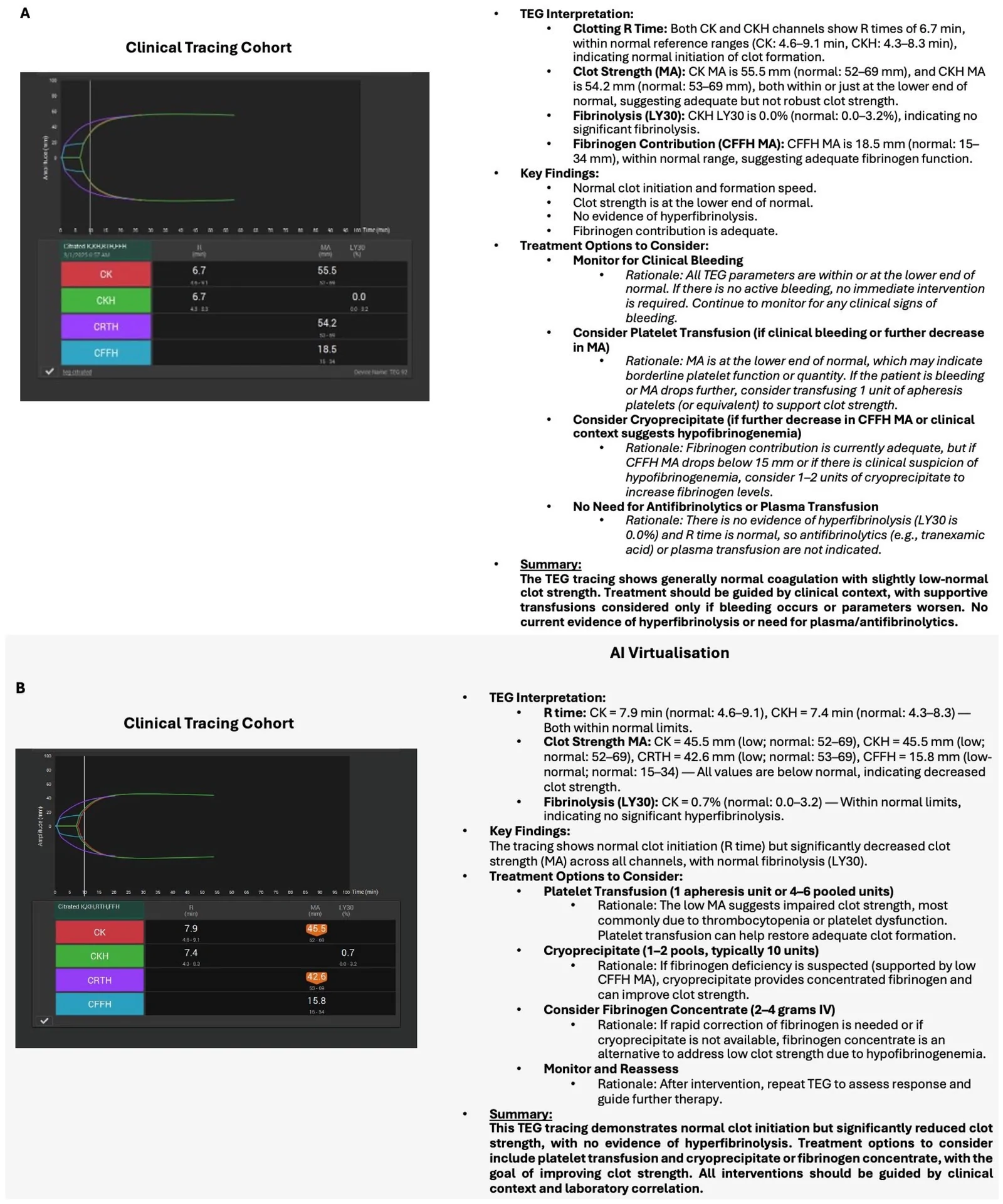

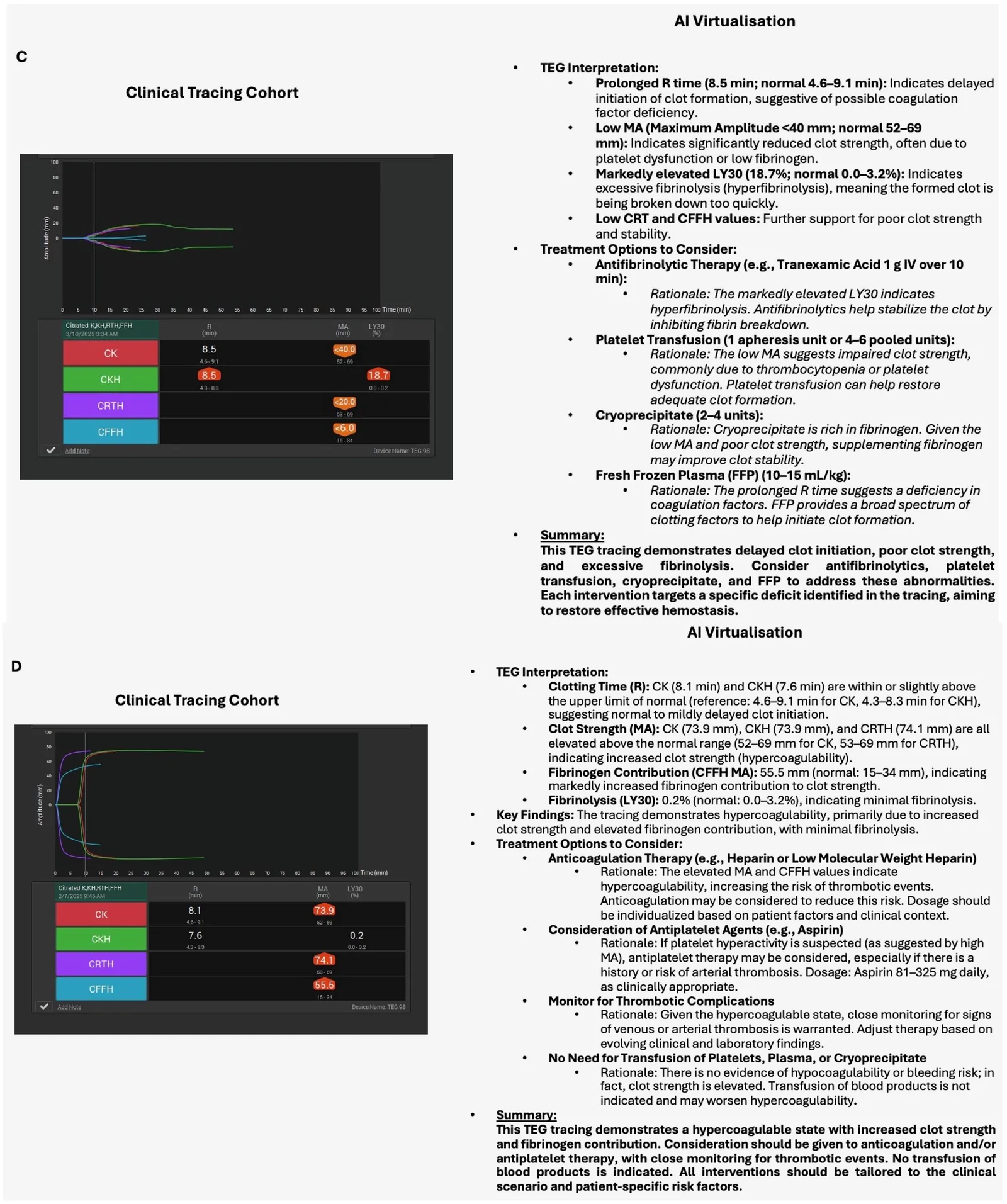

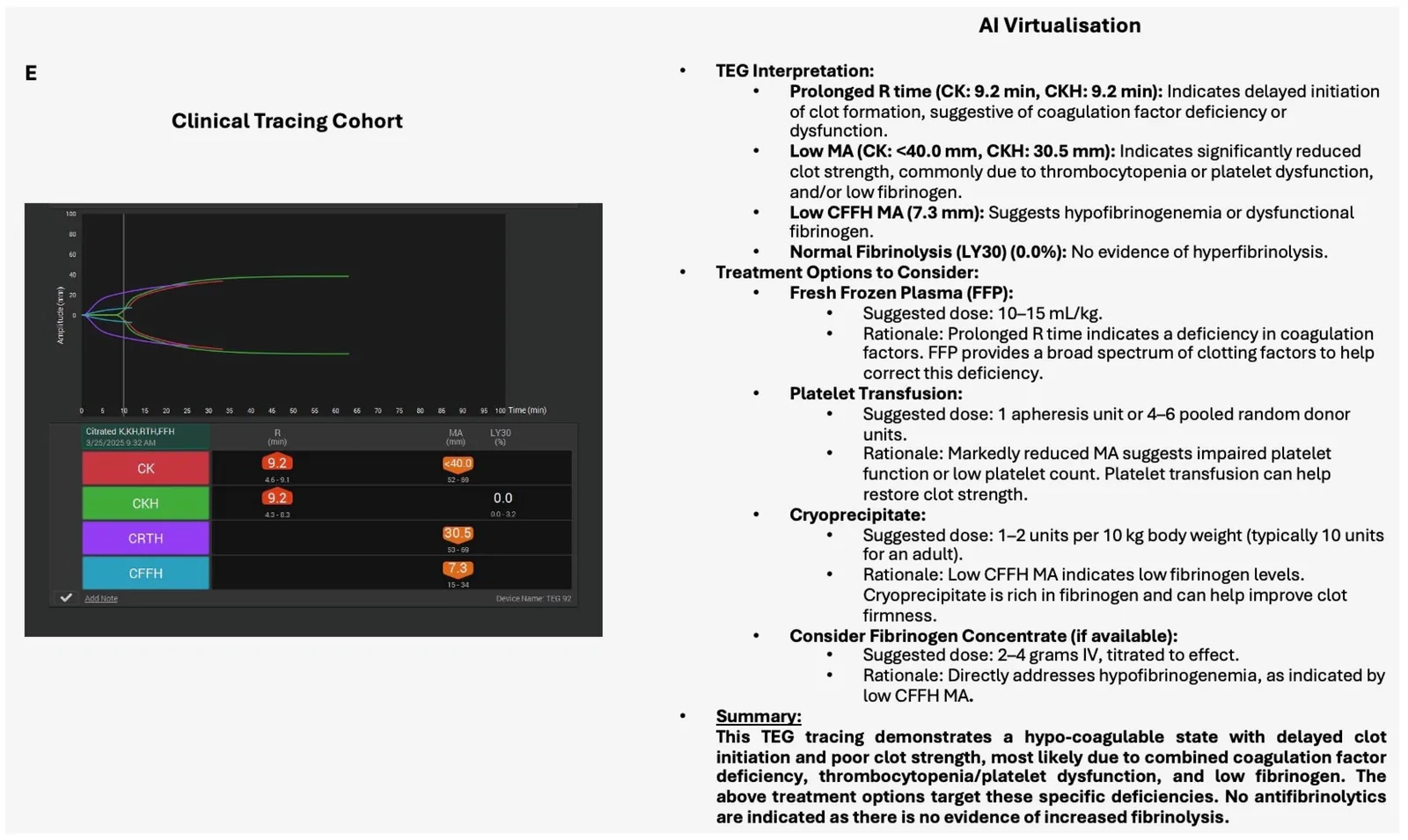
(**A**–**E**) AI virtualization based on clinical tracings. AI, artificial intelligence; CFFH, functional fibrinogen with heparinase; CK, citrated kaolin; CKH, citrated kaolin with heparinase; CRTH, RapidTEG with heparinase; CRT, Citrated RapidTEG without heparinase; FFP, fresh frozen plasma; MA, maximum amplitude; R time, clotting reaction time; IV, intravenous.

Figure 2A–E shows AI virtualization based on clinical TEG^®^ 6s tracings corresponding to five clinical cases. For each tracing, the following parameters are described: a TEG interpretation (in terms of clotting R time, clot strength [MA, maximum amplitude], fibrinolysis at 30 min [LY30], fibrinogen contribution [CFFH, citrated functional fibrinogen with heparinase], and Citrated RapidTEG™ with heparinase [CRTH]) as well as key findings, treatment options to consider, and a summary. The five tests were chosen to train the MetaTEG prototype on real-life situations using several routine patient patterns. These cases represented typical patient patterns encountered in perioperative and critical care settings.

Qualitative descriptive assessments were performed by an internal TEG expert panel specializing in 6s tracing interpretation. They are presented in Table 1. These scores are based on the AI-agent interpretation of TEG^®^ 6s tracings. These are the outcomes of the second round of testing on five TEG^®^ 6s tracings, for which only the latest-generation Heparin Neutralization cartridges were used. Each of the five TEG^®^ 6s tracings was reviewed internally by five TEG experts, whose scores for AI’s diagnostic accuracy, tracing interpretation, and therapeutic recommendations are detailed in Table 1A,B, which shows the average rating per analyzed parameter. The average rating for all tracings is 9.3 (STDEV, 0.6) for tracing interpretation, 9.2 (STDEV, 0.8) for diagnostic accuracy, and 8.2 (STDEV, 1.0) for adequate therapeutic recommendations.

Overall, the AI system received a very good rating, with the scores for all TEG^®^ 6s tracings being quite homogeneous, regardless of the reviewer. The strongest outcome was observed for the descriptive interpretation and the weakest for adequate therapy recommendations. At the same time, however, the standard deviation increased from descriptive interpretation to diagnosis accuracy, and then to adequate therapy recommendations, indicating greater variability between cases. Based on the descriptive evaluation by a TEG expert panel of all three analyzed parameters, the MetaTEG version 1.0 prototype successfully identified TEG^®^ 6s tracings from images, made a diagnosis, and suggested relevant therapeutic recommendations. The AI agent consistently provided real-time clinical decision support suggestions aligned appropriately and accurately with standard treatment protocols.

## 4. Discussion

The use of AI in medicine is growing rapidly and transforming the field by improving diagnostics, patient interaction, and medical forecasting [11]. A recent review described AI-based clinical decision support as a new pathway that is evolving rapidly in terms of the regulation and evaluation of AI medical devices [19]. Previous reports have documented AI use in hemostasis and thrombosis care. As early as 2024, Duranteau et al. already described how the landscape of machine learning models could be mapped to predict transfusions in surgical procedures [20]. Moreover, a recent study reported the successful development of a machine learning model (MLM) incorporating viscoelastic testing and patient-specific variables to predict arterial thrombotic events (ATE) following lower extremity revascularization (LER) after graft or stent thrombosis [21]. Furthermore, a 2025 review reported high accuracy in diagnosing thrombotic events via imaging and electronic health record analysis and described the potential of AI to improve diagnostics, risk prediction, and personalized therapy [22]. Indeed, the idea of using model learning studies regarding VHA interpretation has been around for a while, but a true AI model such as that described here is quite new. Kidal et al. recently reported on a machine learning ROTEM interpretation tool that predicts 30-day mortality when used alongside other clinical predictors of severity [23]. Finally, in their 2022 review, Rashidi et al. described how various machine learning studies in the field of coagulation and hemostasis helped to clarify their shared study requirements, findings, and limitations [24].

To our best knowledge, the MetaTEG version 1.0 prototype is the first generative AI/large language model (LLM) tool specifically trained for TEG^®^ 6s tracing interpretation. The prototype is novel due to its ability to integrate published clinical data with a natural language interface, providing contextual decision support potentially directly at the point of care. Consequently, this MetaTEG prototype mitigates the learning curve associated with TEG interpretation and improves care standardization by eliminating variability introduced through individual user interpretation. Still, physicians remain the critical decision makers, by evaluating the LLM outputs, as part of a human-in-the-loop concept. A very recent review by Scarlatescu et al. describes the current lack of studies using AI to interpret VHAs yet [25]. Noteboom et al. also recently reported that differences between expert evaluations and the ROTEM-guided algorithm underscore the need for advanced clinical decision-making tools; they concluded that future efforts should focus on developing personalized, data-driven algorithms without predefined cutoff values to improve accuracy and patient outcomes [13]. Another study on transfusion prediction supports the potential integration of AI-driven decision support tools into emergency trauma care workflows [26]. Furthermore, AI has been described as offering significant promise for improving diagnostics, risk prediction, and individualized therapy in thrombosis and hemostasis [22].

This proof-of-concept evaluation demonstrated the feasibility of achieving a high level of agreement between AI-generated and expert review interpretations of TEG^®^ 6s tracings, particularly regarding descriptive interpretations and diagnostic categorizations. The highest scores were consistently observed for tracing interpretation, indicating that the AI system reliably identifies key coagulation features within the tracings. This finding is clinically relevant because accurately recognizing coagulation patterns is the first step in viscoelastic-guided management [2]. Notably, the AI-generated outputs included not only numerical classification but also explanatory summaries and contextualized treatment considerations.

The lower scores for therapeutic recommendations compared to tracing interpretation likely reflect the difficulty of translating laboratory findings into individualized treatment decisions. Therapeutic management of coagulation disorders depends not only on VHA results but also on surgical context, active bleeding status, patient comorbidities, institutional transfusion protocols, and physician preference. Consequently, therapeutic recommendations may vary substantially among experienced clinicians despite agreement regarding the underlying coagulation abnormality. The increased standard deviation for treatment recommendations observed in our study supports this interpretation, underlying the multifactorial nature of coagulation management.

Another important aspect of this work is the educational potential of AI-assisted interpretation [27]. Since MetaTEG provides structured explanations in natural language, it can serve as a training tool for clinicians who are unfamiliar with VHA interpretation. This could be particularly beneficial in institutions with limited exposure to TEG technology and in teaching hospitals, where frequent staff turnover necessitates repeated educational efforts. AI-assisted interpretation could complement traditional training approaches by reinforcing standardized interpretation frameworks and promoting consistency among providers [10,28]. The human-in-the-loop approach remains central to the intended use of MetaTEG. Rather than replacing physician judgment, MetaTEG is designed to augment clinician interpretation by rapidly synthesizing complex tracing information and generating contextual recommendations. Maintaining physician oversight is important, especially given the current limitations of generative AI systems. These limitations include incomplete reasoning, overgeneralization, and hallucinated outputs [27]. Some potential risks associated with LLM-based clinical decision support systems have emerged, such as hallucinations, incorrect recommendations, automation bias, and overreliance on AI-generated outputs, highlighting the continued need for human oversight and rigorous validation [10,29]. In this context, MetaTEG should be considered a clinical decision support tool rather than an autonomous diagnostic system.

Another notable aspect of the prototype is its use of multimodal AI architecture. The MetaTEG framework combines image-based tracing interpretation with text-based reasoning and retrieval of clinical knowledge sources. Unlike conventional rule-based systems, this approach enables the contextual interpretation of graphical waveform information and associated coagulation parameters. Future iterations may expand this capability further by integrating additional patient-specific variables, such as laboratory values, transfusion history, anticoagulant therapy, and procedural context. Such integration could support increasingly personalized approaches to coagulation management. The average descriptive ratings are in line with typical expectations for in vitro diagnostics (IVDs), since diagnosing a disease is generally easier than determining the appropriate therapy. Reasons for the lower therapy scores may include regional variations in medical practice and possible limitations in the model’s training data.

As MetaTEG version 1.0 is an early prototype, our study has several limitations. These include the limited sample size and the restricted variety of presented cases, underscoring the need for broader clinical validation using more diverse datasets, patient populations, and cartridge types to ensure reliability and accuracy across different clinical settings. Although the descriptive rating was based on a quantitative scale, interpretation was left to individual reviewers, each applying their own judgment and rating philosophy. This could have introduced subjectivity, particularly because the scoring system was not standardized or validated. The reviewers were experts in TEG and viscoelastic testing with substantial technical and scientific expertise. However, their direct clinical experience with viscoelastic testing in real-world patient care was limited, which may have influenced certain aspects of the evaluation. In addition, all reviewers were internal to the manufacturer; while this ensured deep familiarity with the technology, it may also have introduced bias into the assessment.

Several additional limitations should be noted. First, the study evaluated static, image-based tracings rather than complete clinical cases with integrated laboratory and patient information. Consequently, the therapeutic recommendations generated by the AI system were based on limited contextual information. Second, the current prototype focused specifically on the Global Hemostasis-Heparin Neutralization cartridge; therefore, it may not be applicable to all TEG^®^ assays or alternative VHA platforms. Third, the study did not compare MetaTEG directly with nonexpert clinicians or alternative interpretation methods, which limits conclusions regarding comparative performance. Fourthly, as this initial proof-of-concept evaluation did not use a standardized or validated expert scoring system, the outcomes cannot be generalized, should be treated with caution, and viewed as a trend to inform further analysis with larger samples and a scoring system.

Attention was given to workflow integration and usability. The prototype interface was designed to enable quick interpretation in acute clinical settings where time-sensitive decisions are necessary. The display architecture prioritizes visual clarity, combining the original tracing image with concise interpretive summaries and suggested treatment considerations. Potential future applications could include integration into perioperative dashboards, transfusion management pathways, and electronic medical record systems. While the current version is still a research prototype, the design process aimed to simulate realistic clinical workflows and identify features that could facilitate future adoption at the bedside. Therefore, future studies should include prospective, multicenter evaluations involving external reviewers and larger datasets representing broader patient populations and coagulation abnormalities. Additional validation against real-world clinical outcomes is also important for determining whether AI-assisted interpretation can improve transfusion management, reduce unnecessary blood product utilization, and enhance adherence to evidence-based coagulation algorithms [28]. Furthermore, regulatory considerations and cybersecurity safeguards will be critical to the implementation of AI-enabled clinical decision support systems [27].

Despite these limitations, the current findings demonstrate the feasibility of applying generative AI technologies to viscoelastic hemostatic assay interpretation. The study also highlights the growing convergence between digital health technologies, laboratory medicine, and perioperative decision support [28,29,30]. As AI tools evolve, their role in supporting rapid, standardized interpretation of complex diagnostic data will likely expand substantially.

## 5. Conclusions

This analysis demonstrates the initial feasibility of the MetaTEG prototype for providing AI-powered decision support for TEG^®^ 6s tracings. Following evaluation by a TEG expert panel, this report provides proof of concept for the validity of the results obtained using the MetaTEG prototype. This research tool offers potential development opportunities for improving efficiency and standardizing care through further development. Importantly, the findings suggest that generative AI can support diagnostic interpretation and educational reinforcement in viscoelastic hemostatic testing. MetaTEG is a novel approach to integrating AI-assisted reasoning into coagulation management workflows because it combines tracing recognition with contextualized natural language explanations. The next steps include planning formal validation studies and exploring strategies to integrate the MetaTEG model into clinical workflows. Given the transformative potential of AI in healthcare, large-scale clinical studies are warranted to further train, validate, and generalize the MetaTEG model across different patient populations and care settings. Future development efforts should evaluate the integration of electronic medical records, workflow automation, and prospective, outcome-based studies that assess the clinical utility of AI-assisted viscoelastic interpretation.

## 6. Patents

A provisional patent application has been filed related to the methods described in this study.

## Figures and Tables

**Table 1:** Qualitative descriptive scoring by an internal TEG^®^ 6s tracing expert panel on AI-agent interpretation, with a focus on the global hemostasis-heparin neutralization cartridge. (**A**) Detailed ratings. (**B**) Average ratings.

(A)							
**Reviewers’ Rating**		TEG^®^ 6s Tracings					
**Reviewer #1**		**#1**	**#2**	**#3**	**#4**	**#5**	
Tracing interpretation		10	10	10	10	10	
Diagnostic accuracy		10	8	9	9	10	
Adequate therapeutic recommendations		9	9	8	6	9	
**Reviewer #2**		**#1**	**#2**	**#3**	**#4**	**#5**	
Tracing interpretation		10	10	9	9	10	
Diagnostic accuracy		10	10	9	9	10	
Adequate therapeutic recommendations		10	9	8	6	9	
**Reviewer #3**		**#1**	**#2**	**#3**	**#4**	**#5**	
Tracing interpretation		10	10	6	8	9	
Diagnostic accuracy		10	8	7	8	9	
Adequate therapeutic recommendations		7	8	7	8	9	
**Reviewer #4**		**#1**	**#2**	**#3**	**#4**	**#5**	
Tracing interpretation		10	10	9	9	10	
Diagnostic accuracy		10	10	7	9	10	
Adequate therapeutic recommendations		10	9	6	6	8	
**Reviewer #5**		**#1**	**#2**	**#3**	**#4**	**#5**	
Tracing interpretation		9	9	9	8	9	
Diagnostic accuracy		10	10	9	8	10	
Adequate therapeutic recommendations		9	9	9	8	9	
(**B**)							
**Reviewers’ Ratings, Average (Range)**	**TEG** ^®^ **6s Tracings**					**Total**	
**All reviewers**	**#1**	**#2**	**#3**	**#4**	**#5**	**All Tracings**	**STDEV**
Tracing interpretation	9.8	9.8	8.6	8.8	9.6	9.3	0.6
	(9–10)	(9–10)	(6–10)	(8–10)	(9–10)		
Diagnostic accuracy	10.0	9.2	8.2	8.6	9.8	9.2	0.8
	(10–10)	(8–10)	(7–9)	(8–9)	(9–10)		
Adequate therapeutic recommendations	9.0	8.8	7.6	6.8	8.8	8.2	1.0
	(7–10)	(8–9)	(6–9)	(6–8)	(8–9)		

STDEV, standard deviation.

## Data Availability

The original contributions presented in this study are included in the article. Further inquiries can be directed to the corresponding author.

## Abbreviations

The following abbreviations are used in this manuscript:

AI	Artificial intelligence
ATE	Arterial thrombotic events
CFF(H)	Citrated functional fibrinogen (with heparinase)
CK(H)	Citrated kaolin (with heparinase)
CRT(H)	Citrated RapidTEG (with heparinase)
FFP	Fresh frozen plasma
IV	Intravenous
IVDs	in vitro diagnostics
LER	Lower extremity revascularization
LLM	Large language model
MA	Maximum amplitude
MLM	Machine learning model
NLP	Natural language processing
R time	Clotting reaction time
TEG	Thromboelastography
VHA	Viscoelastic hemostatic assay
